# Direct measurement of Stokes–Einstein diffusion of Cowpea mosaic virus with 19 µs-resolved XPCS

**DOI:** 10.1107/S1600577522008402

**Published:** 2022-10-10

**Authors:** Kacper Switalski, Jingyu Fan, Luxi Li, Miaoqi Chu, Erik Sarnello, Pete Jemian, Tao Li, Qian Wang, Qingteng Zhang

**Affiliations:** a Department of Chemical Engineering, University of Illinois at Chicago, Chicago, IL 60611, USA; b Department of Chemistry and Biochemistry, University of South Carolina, Columbia, SC 29208, USA; c X-ray Science Division, Argonne National Laboratory, 9700 South Cass Avenue, Argonne, IL 60439, USA; d Department of Chemistry and Biochemistry, Northern Illinois University, DeKalb, IL 60115, USA; University of Tokyo, Japan

**Keywords:** XPCS, SAXS, biomolecules, virus-like particles (VLPs), high-speed X-ray detectors

## Abstract

Brownian motion of Cowpea mosaic virus (CPMV) in water was measured using small-angle X-ray photon correlation spectroscopy (SA-XPCS) at 19.2 µs time resolution. It was found that the decorrelation time τ(*Q*) = 1/*DQ* ^2^ up to *Q* = 0.091 nm^−1^. The hydrodynamic radius *R*_H_ determined from XPCS using Stokes–Einstein diffusion *D* = *kT*/(6πη*R*_H_) is 43% larger than the geometric radius *R*_0_ determined from SAXS in the 0.007 *M* K_3_PO_4_ buffer solution, whereas it is 80% larger for CPMV in 0.5 *M* NaCl and 104% larger in 0.5 *M* (NH_4_)_2_SO_4_, a possible effect of aggregation as well as slight variation of the structures of the capsid resulting from the salt–protein interactions.

## Abstract 2

Stokes–Einstein diffusion of active Cowpea mosaic virus was measured up to a 69 nm length-scale with 19 µs-resolved X-ray photon correlation spectroscopy.

## 1. Introduction

X-ray photon correlation spectroscopy (XPCS) is a coherent X-ray scattering technique that directly probes the dynamic structure factor *S*(*Q*, ω) in condensed matter. This is done by measuring the intensity autocorrelation function *g*_2_(τ, *Q*) from coherent X-ray scattering intensities (‘speckles’). Aside from the time-averaged static structure factor *S*(*Q*) provided by X-ray scattering (*e.g.* small-angle X-ray scattering, SAXS), XPCS also provides the fluctuation time scale of *S*(*Q*) similar to dynamic light scattering (DLS). The use of a hard X-ray beam with sub-Ångstrom wavelength not only allows XPCS to probe optically opaque samples (Yavitt *et al.*, 2021 ▸) with sophisticated *in situ* (Ju *et al.*, 2019 ▸) and *operando* (Lin *et al.*, 2021 ▸) sample environments, but also provides spatial sensitivity to structural fluctuation over a wide range of length scales, *i.e.* from sub-micrometre (Dallari *et al.*, 2020 ▸) to tens of picometres (Ruta *et al.*, 2020 ▸).

The rapid emergence of next-generation X-ray sources, including near diffraction-limited storage rings (DLSRs) such as PETRA IV (Schroer *et al.*, 2018 ▸), MAX IV (Björklund Svensson *et al.*, 2019 ▸), ESRF–EBS (Chenevier & Joly, 2018 ▸) and APS-U (Dooling *et al.*, 2022 ▸), as well as free-electron lasers (FELs) such as European XFEL (Tschentscher *et al.*, 2017 ▸), LCLS II (Halavanau *et al.*, 2019 ▸), SwissFEL (Milne *et al.*, 2017 ▸) and SACLA (Yabashi *et al.*, 2017 ▸), promises an increase of coherent X-ray flux by several orders of magnitude. Combined with the development of high-speed, high-fidelity pixelated photon-counting X-ray detectors (Pennicard *et al.*, 2018 ▸; Allahgholi *et al.*, 2019 ▸; Ballabriga *et al.*, 2018 ▸; Möller *et al.*, 2019 ▸), XPCS will have the potential to fill the ‘no-man’s land’ in *S*(*Q*, ω) from 0.1 nm to 100 nm and 10^4^ Hz to 10^8^ Hz (Shpyrko, 2014 ▸). The advance of the temporospatial scales of XPCS will not only extend from the well established works of Brownian motions in colloidal suspensions (Fluerasu *et al.*, 2010 ▸; Urbani *et al.*, 2016 ▸; Caronna *et al.*, 2008 ▸; Möller & Narayanan, 2017 ▸; Pal *et al.*, 2018 ▸; Ragulskaya *et al.*, 2022 ▸), but also expand the scope to cover non-equilibrium dynamics during phase separations, including micelles (Sheyfer *et al.*, 2020 ▸) and macromolecules such as domain-forming (Girelli *et al.*, 2021 ▸) and free-diffusing (Vodnala *et al.*, 2018 ▸) protein suspensions.

**Figure 1:**
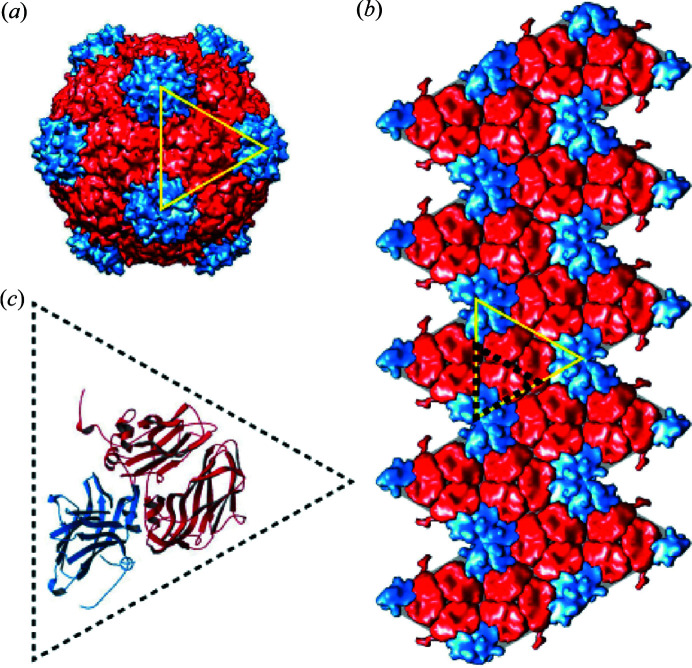
(*a*) 3D illustration of the icosahedral capsid of CPMV. (*b*) Flattened layout of the icosahedral capsid. The yellow triangles in (*a*) and (*b*) represent the same surface. (*c*) Two types of proteins (red and blue) that form one of the 60 units (black dashed triangle), as shown in (*b*). The figures were obtained from *VIPERdb* (http://viperdb.scripps.edu) (Montiel-Garcia *et al.*, 2021 ▸).

One of the simplest and biologically relevant hydrodynamic scenarios in condense matter is the diffusion of viruses and virus-like particles. Viruses are typically monodisperse with a <100 nm geometric radius *R*_0_ and are incapable of self-propelled motion (Tejeda-Rodríguez *et al.*, 2019 ▸). The dynamics of dilute virus suspension in aqueous environments is therefore speculated to behave largely similar to Brownian motion (Hammermann *et al.*, 1997 ▸; Song *et al.*, 1991 ▸). Cowpea mosaic virus (CPMV) is a non-enveloped, icosahedral-shaped virus with a radius of ∼15 nm. The genome RNA of CPMV is surrounded by its capsid, a spherical shell comprising 60 identical units each consisting of two types of protein (Fig. 1 ▸). CPMV is an ideal model for virus-like particles in this study because (1) CPMV can be readily harvested and purified in gram quantities (Wang *et al.*, 2002 ▸); (2) the molecular structure of CPMV is known to sub-nanometre precision (Lin *et al.*, 1999 ▸); (3) CPMV can be engineered via genetic mutations (Johnson *et al.*, 1997 ▸) and chemical modification with high selectivity (Strable *et al.*, 2004 ▸; Souza *et al.*, 2002 ▸; Wang *et al.*, 2002 ▸), making it useful as a template for hybridized nanomaterials (Uchida *et al.*, 2007 ▸), vehicles for targeted drug delivery (Beatty & Lewis, 2019 ▸) and scaffolds for vaccine development (Lizotte *et al.*, 2016 ▸; Miermont *et al.*, 2008 ▸).

Here we demonstrate the small-angle XPCS (SA-XPCS) measurement on the hydrodynamics of dilute CPMV suspension in aqueous environments. Our SAXS results yield a geometric radius *R*_0_ of 13.0 nm for CPMV, consistent with previous literature (Lin *et al.*, 1999 ▸). However, the hydrodynamic radius *R*_H_, determined by directly measuring the diffusion coefficient using XPCS, is 18.7 ± 0.7 nm in the buffer solution (0.007 *M* K_3_PO_4_), 23.4 ± 1.6 nm with an additional 0.5 *M* NaCl and 26.5 ± 1.3 nm with an additional 0.5 *M* (NH_4_)_2_SO_4_. The difference of *R*_H_ in different salt solutions may arise from a combination of factors including ionic strength and effects from the Hofmeister series. The remainder of this paper is organized as follows: Section 2 describes the preparation of the CPMV samples; Section 3 outlines the instrumental conditions for SA-XPCS measurements; Sections 4 and 5 summarize the SAXS and XPCS results which leads to the evaluation of *R*_0_ and *R*_H_, respectively; Section 6 examines the differences between *R*_0_ and *R*_H_ under different ionic strengths and salt types; and Section 7 provides an outlook of the scientific opportunities that can be enabled by microsecond-resolved XPCS and ultra-high brilliance of the coherent X-ray beams of next-generation light sources.

## 2. CPMV sample preparation

Cowpea plants approximately 1 month old were inoculated with CPMV. The leaves from the host plant were crushed and added to 0.01 *M* K_3_PO_4_ buffer at pH 7.8 with 0.2% mercapto­ethanol. The mixture was centrifuged at 9000 rpm for 15 min and the supernatant was treated with a 1:1 ratio of CHCl_3_:1-butanol. The aqueous portion was separated and CPMV was precipitated by adding polyethylene glycol 8 K and NaCl. The resultant pellets were resuspended in 0.01 *M* K_3_PO_4_ buffer at pH 7.8. After a final ultracentrifugation at 42000 rpm for 2.5 h, pure CPMV was obtained and then resuspended overnight in 0.1 *M* K_3_PO_4_ buffer at pH 7.8 or in deionized water to produce the stock sample with 10.96 mg ml^−1^ CPMV concentration. The stock solution was aliquoted and mixed at a 7:1 ratio with deionized water, 4 *M* NaCl solution and 4 *M* (NH_4_)_2_SO_4_, respectively, to produce three sample conditions with identical CPMV concentrations (9.59 mg ml^−1^) but with 0.007 *M* K_3_PO_4_, 0.007 *M* K_3_PO_4_ + 0.5 *M* NaCl, and 0.007 *M* K_3_PO_4_ + 0.5 M (NH_4_)_2_SO_4_, hereafter referred to as samples A, B and C. The samples were then pipetted into 40 mm-long, 2 mm-diameter thin-walled quartz capillary tubes (Charles–Supper) and fitted into customized aluminium blocks to maintain the sample temperature at 6°C throughout the SA-XPCS measurements.

## 3. SA-XPCS beamline instrumentation

The SA-XPCS measurements were performed at station 8-ID-I of the Advanced Photon Source at Argonne National Laboratory. The X-ray beam was generated from a tandem of 33 mm-period, 2.4 m-long undulators. The beam was then deflected by a plane silicon mirror at a 5 mrad angle to remove the higher harmonics, and passed through a Ge(111) monochromator with a 0.03% relative bandpass to select a longitudinally coherent X-ray beam with a photon energy of 10.94 keV. For transverse coherence, a 180 µm (vertical) × 15 µm (horizontal) portion of the beam was selected by tungsten-blade guard slits and then focused vertically using 15 pieces of beryllium compound refractive lenses (CRLs). The final beam footprint on the sample is 15 µm (horizontal) × 10 µm (vertical) with a flux of 1.2 × 10^10^ photons s^−1^.

The transmitted coherent X-ray scattering intensities were collected 8 m downstream of the sample using X-ray Seamless Pixel Array 500k (XSPA-500k), a single-photon-counting detector with a pixel size of 76 µm and tunable frame rate up to 52 kHz (Nakaye *et al.*, 2021 ▸). For the current study, the frame rate was fixed at 52 kHz and each measurement consisted of 100000 detector frames collected continuously over a total duration of 1.92 s. Due to the extremely low scattering rate of CPMV, each individual measurement of 100000 frames was further repeated 14328 times for sample A, 7554 times for sample B and 13054 times for sample C, resulting in approximately one billion detector frames for each sample condition. The results from repeated measurements were averaged to produce *g*_2_(τ, *Q*) with sufficient statistics for quantitative analysis. As a side note, to facilitate peer communication and community growth, the entire data life-cycle of this study has been made 100% open-source: (1) all SA-XPCS measurements were performed using *Bluesky*, a Python-based beamline control system (Arkilic *et al.*, 2017 ▸); (2) the sparsified detector frames (∼25 TB) were transferred and analyzed in near real-time using the APS *Data Management* workflow (Veseli *et al.*, 2018 ▸); (3) the SA-XPCS results for each sample condition were averaged and visualized in *pyXpcsViewer* (Chu *et al.*, 2022 ▸); (4) the reduced results were analyzed using pyXpcsViewer script mode and plotted using *Matplotlib* in a *JupyterLab* environment. Full 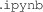 files including data analysis, figure rendering and the figures (embedded inline) can be found on GitHub (Zhang, 2022 ▸); (5) the manuscript was prepared in *Overleaf*. More details regarding the SAXS and XPCS methods in this study can be found in Sections 4 and 5.

## 4. SAXS and *R*_0_

**Figure 2:**
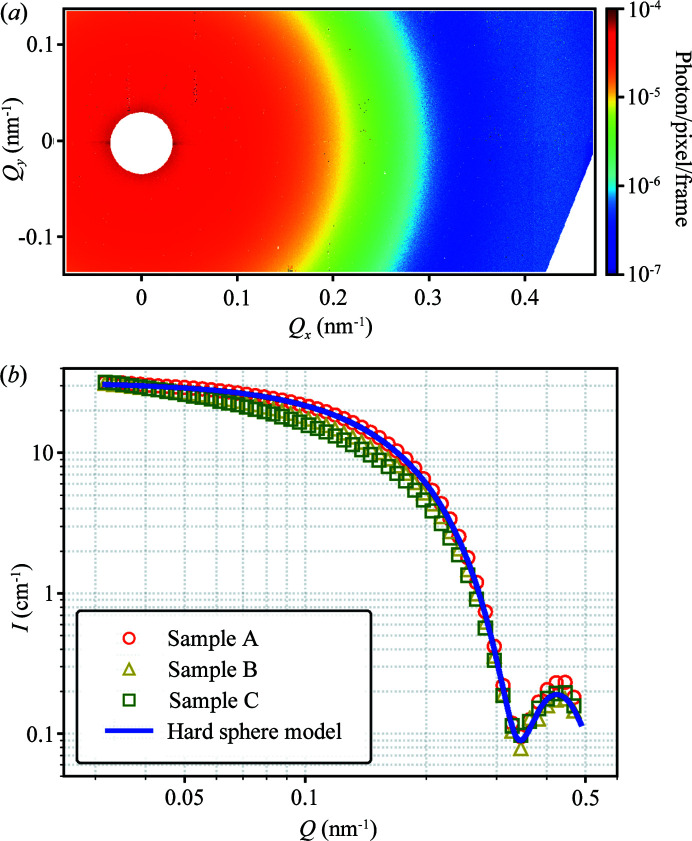
(*a*) SAXS from 9.59 mg ml^−1^ CPMV suspension in 0.007 *M* K_3_PO_4_ buffer (sample A) averaged over 100000 frames collected continuously within 1.92 s. The result was further averaged from 1000 repeated measurements to improve the statistics. (*b*) Azimuthal average of (*a*) for sample A (red circles), 9.59 mg ml^−1^ CPMV in 0.007 *M* K_3_PO_4_ and 0.5 *M* NaCl (sample B, yellow triangle), 9.59 mg ml^−1^ CPMV in 0.007 *M* K_3_PO_4_ and 0.5 *M* (NH_4_)_2_SO_4_ (sample C, green square). The solid blue line is the form factor calculated from spherical particles with a Gaussian distribution of the radius (average = 13.0 nm, standard deviation = 1.2 nm).

Fig. 2 ▸(*a*) shows the 2D SAXS measured from sample A, where the 100000 frames acquired at a 52 kHz frame rate are averaged over time to produce the equivalent of an SAXS measurement from a single 1.92 s exposure. The 2D SAXS is then further averaged over 1000 repeating measurements to improve the statistics. The white circular region in Fig. 2 ▸(*a*) is the shadow from an ∼3 mm-diameter tungsten cylinder placed ∼10 cm in front of the detector to block the direct beam (*i.e.* beamstop), and the white triangular region at the bottom right corner is the cutoff from the downstream rim of the 8 m-long vacuum tube (*i.e.* flight path). Both regions were masked out and excluded from the SAXS and XPCS analyses. Fig. 2 ▸(*b*) shows the 1D SAXS azimuthally averaged from the 2D SAXS in Fig. 2 ▸(*a*), where the pixels in Fig. 2 ▸(*a*) were grouped into 270 logarithmically spaced partitions of *Q* and the scattering intensities were averaged within each partition. The form factor of CPMV is consistent with the prediction from nanospheres with a Gaussian size distribution (Rieker *et al.*, 1999 ▸), yielding a geometric radius *R*_0_ of 13.0 ± 1.2 nm, consistent with the range of 12.7 nm (twofold axis) to 15.4 nm (fivefold axis) for the icosahedral CPMV structure measured using X-ray diffraction (Lin *et al.*, 1999 ▸). For samples B and C, signs of aggregation can be observed from the tilting of the 1D SAXS at the lower *Q* region; however, the change is slightly more pronounced in sample C than sample B. Besides the fact that the ionic strength in sample C is three times higher than sample B, we also notice that, in the Hofmeister series, both the cation and the anion of (NH_4_)_2_SO_4_ in sample C are ranked higher than the cation and anion of NaCl in sample B. We therefore suspect that the nuanced difference in aggregation may be attributed to both the higher level of compensation of the static charge on the capsid surface as well as the slight modification to the structures of the capsid.

## 5. XPCS and *R*_H_

**Figure 3:**
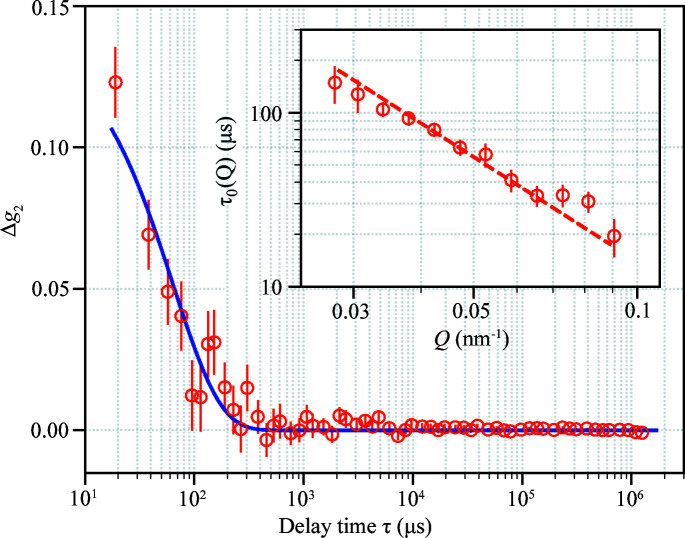
Intensity autocorrelation function Δ*g*_2_ = *g*_2_ − 1 averaged over 14328 repeated measurements from sample A. The blue solid line shows the fitting of Δ*g*_2_ = βexp[−2τ/τ_0_(*Q*)]. The inset shows the fit with the Stokes–Einstein equation τ_0_(*Q*) = 1/(*DQ* ^2^) (red dashed line).

The difference in the ionic strength and salt type among samples A, B and C is more pronounced in the Stokes–Einstein diffusion of CPMV as directly probed by XPCS. Fig. 3 ▸ shows Δ*g*_2_ = *g*_2_ − 1 (in the absence of correlation, *g*_2_ = 1) at *Q* = 0.031 nm^−1^ from sample A, where *g*_2_(τ, *Q*) is calculated using the following method (Zhang *et al.*, 2018 ▸),

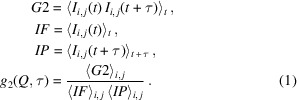

Here, *t* and *t* + τ are the measurement time of detector frames within the 100000 frame sequence and τ is the delay time between the two frames. The time average 〈…〉_*t*_ and 〈…〉_*t*+τ_ go from 0 to *T* − τ and τ to *T*, respectively, where time 0 and *T* are the start and end times of the frame sequence. In case the scattering intensity does not vary within the measurement (*e.g.* Fig. S1 of the supporting information), *IP* and *IF* are invariant of τ and are equal to the 2D SAXS pattern in Fig. 2 ▸(*a*). When evaluating *G*2, *IF* and *IP* at larger delay time τ, the frames are binned in an exponentially recursive manner based on the *multi-tau* algorithm used in DLS (Figs. S3 and S4).

In the spatial regime, *i* and *j* are pixel indices and *Q* denotes the momentum transfer of the region where pixel binning 〈...〉_*i*,*j*_ is performed. In equation (1), the pixel binning 〈...〉_*i*,*j*_ is first performed using the same azimuthal averaging method that converts 2D SAXS [Fig. 2 ▸(*a*)] to 1D SAXS [Fig. 2 ▸(*b*)] with a width of approximately 2 pixels (Δ*Q* ≃ 1 × 10^−3^ nm), and the resulting *g*_2_ is further binned by a factor of 10 in *Q* (Δ*Q* ≃ 1 × 10^−2^ nm) to improve the overall signal to noise ratio. Use of initially narrower regions of pixel binning in the *g*_2_ calculation reduces intensity variation from 1D SAXS within the binning region, which is known to increase the *g*_2_ baseline as detailed in previous studies (Sheyfer *et al.*, 2020 ▸). Note that the pixel binning is performed on *G*2, *IF* and *IP* instead of *g*_2_-per-pixel, *i.e.* pixel-wise division of *G*2/(*IF* × *IP*). Performing the pixel binning before the division allows for evaluation of the coherence factor β (explained later in Fig. 3 ▸) in the absence of temporal decorrelation, a quantity similar to fringe visibility in a multi-slit diffraction with a visible laser. The error in *g*_2_ is determined as the standard deviation of *g*_2_-per-pixel within the larger Δ*Q* ≃ 1 × 10^−2^ nm region on the detector where *g*_2_(*Q*, τ) is determined.

Typically, for samples with low scattering rates, *g*_2_ is determined for each individual measurement and then averaged over repeating measurements to improve the statistics (Zhang *et al.*, 2021 ▸). However, due to the extremely low scattering rate of the CPMV samples (∼7 × 10^−5^ photon per pixel per detector frame), *G*2, *IF* and *IP* were averaged first before determining *g*_2_ and the error using equation (1). The averaged *G*2 has sufficient statistics to help identify noisy pixels that are too subtle to be flagged from the averaged scattering intensity, *e.g.* pixels with abnormally high correlation values due to the overlap in the gating signals of the double-counters on the pixel (Zhang *et al.*, 2016 ▸), an artifact whose impact on *g*_2_ is inversely proportional to the count rates.

**Figure 4:**
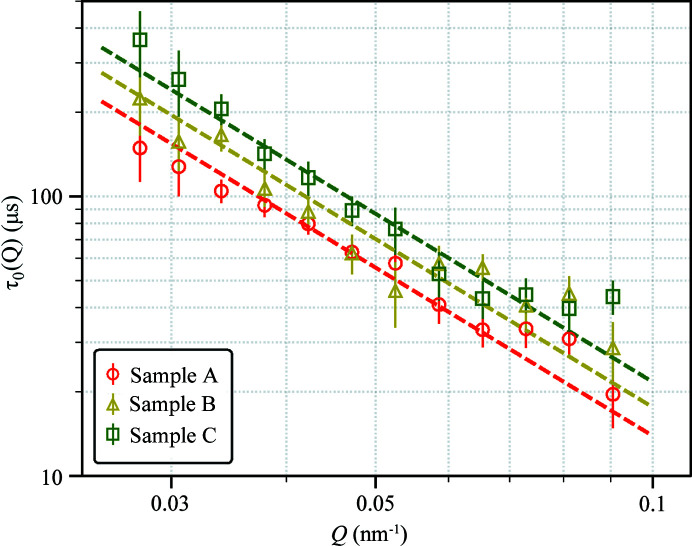
Comparison of CPMV diffusivity in samples A, B and C. The dashed lines represent the best fit to τ_0_(*Q*) = 1/(*DQ* ^2^) for each sample condition.

The dynamic time scale τ_0_(*Q*) of CPMV colloidal suspension at various length scales is evaluated by fitting Δ*g*_2_(τ, *Q*) at different *Q* values with a simple exponential function Δ*g*_2_(τ, *Q*) = βexp[−2τ/τ_0_(*Q*)] (solid blue line in Fig. 3 ▸), where β = 0.14 is the coherence factor of the beamline and was determined from the *g*_2_(τ, *Q*) of a static reference sample. We notice that τ_0_(*Q*) follows the prediction from the Stokes–Einstein equation of 1/(*DQ* ^2^) up to ∼0.1 nm^−1^, as shown by the dashed red line in the inset of Fig. 3 ▸. The hydrodynamic radius *R*_H_ of CPMV was determined from the diffusivity *D* = *kT*/(6πη*R*_H_), where *k* is the Boltzmann constant, *T* = 279 K and η = 1.520 × 10^−3^ Pa s is the viscosity of water at 6°C (Kestin *et al.*, 1978 ▸). We found *R*_H_ = 18.7 ± 0.7 nm for sample A, which is 43% larger than *R*_0_ = 13.0 ± 1.2 nm determined from SAXS. Fig. 4 ▸ shows the Stokes–Einstein diffusion measured from samples A, B and C. Taking into account the increase of water viscosity in the presence of electrolytes using the Jones–Dole model (Jenkins & Marcus, 1995 ▸), η = 1.536 × 10^−3^ Pa s for sample B and η = 1.672 × 10^−3^ Pa s for sample C, which lead to *R*_H_ = 23.4 ± 1.6 nm for sample B and *R*_H_ = 26.5 ± 1.3 nm for sample C, respectively. The effect of *R*_H_ > *R*_0_ is therefore more pronounced in solution with higher ionic strength and in (NH_4_)_2_SO_4_ than NaCl.

## 6. Discussion

Although the diffusion of CPMV follows Stokes–Einstein equations, we noticed that *R*_H_ is larger than *R*_0_ for all solvent conditions considered. Consistency among SAXS and XPCS results from subsets of 100000-frame acquisitions (Figs. S1 and S2, respectively) indicates there is no observable radiation damage. In addition, at a 0.7% CPMV volume fraction, the hydrodynamic collective interactions, as seen in more concentrated colloidal suspensions (Robert *et al.*, 2008 ▸; Orsi *et al.*, 2012 ▸), may not be significant enough to account for the observed dynamic behavior. However, given the minimum detectable *Q* ≃ 0.03 nm^−1^, we cannot rule out the formation of larger scale aggregation that may slow down CPMV hydrodynamics as measured in XPCS. Another possible explanation for the larger *R*_H_ in sample A is the electrostatic repulsion from the negative charge on the C-terminal peptide of the S-coat protein (Meshcheriakova & Lomonossoff, 2019 ▸). Similar static charge has been observed in a variety of capsids, including Cowpea chlorotic mottle virus (Lucas *et al.*, 2002 ▸; Liu *et al.*, 2012 ▸) which belongs to the same Bromovirus genus, Tobacco mosaic virus (Bendahmane *et al.*, 1999 ▸) and spherical protein cages like apoferrtin (Garmann *et al.*, 2014 ▸; Böker *et al.*, 2007 ▸). Although the mechanism that resulted in further increase of *R*_H_ in samples B and C remains unclear, we postulate that, besides the increase of ionic strength, one possible mechanism could be the Hofmeister effects of different ions (Kunz *et al.*, 2004 ▸). It is well known that adding salts to protein aqueous solution has significant impacts on the solubility and other physiochemical properties of the protein solution. Generally, SO_4_^2−^ and NH_4_^+^ can decrease the solubility of proteins (*i.e.* the ‘salt-out’ process) much more strongly that Na^+^ and Cl^−^. As early members of the Hof­meister series, SO_4_^2−^ and NH_4_^+^ can increase the surface tension of the solution and strengthen the hydrophobic interaction of proteins much more significantly than later members like Na^+^ and Cl^−^. On the other hand, we cannot overlook the direct ion–protein interactions as well as interactions of ions with water molecules in the first hydration shell of the macromolecule as illustrated by many recent studies (Zhang & Cremer, 2006 ▸). In the case of CPMV, three major interactions will contribute to its hydrodynamic properties: (1) interactions of single coat proteins with aqueous media, (2) interactions between neighboring coat proteins and (3) attractions between the coat protein shell and the genomic RNA core. As a result, it is extremely difficult to untangle the complex effects of ions to all three interactions and to understand how those interactions influence the *R*_H_ of CPMV particles in aqueous environments, especially given the ion-specificity effects that play a critical role in the biological and physiological behaviors of biomacromolecules and viruses. XPCS therefore provides a direct analytical tool to monitor the hydrodynamic properties of nanoscale particles which can help us to uncover the complexity of ion-specific effects on virus and other nanoscale bioassemblies.

## 7. Outlook

Biomacromolecules present richer tunabilities in their structural and dynamic properties compared with inorganic nanoparticles because the very structure of the molecules can be altered either by design (genetic sequence) or by environment (temperature, ionic strength *etc*.). These changes can trigger phase transitions, where reconfiguration of molecular structures leads to increased interaction strength and causes biomacromolecules to self-assemble into mesoscale domains or fractals, eventually resulting in macroscopic structures with a wide range of porosity, viscosity, elasticity, opacity *etc*. Such non-equilibrium dynamics are characterized by their telescopic length scales, rapid fluctuation and constantly evolving dynamics, which can be technically challenging for raster imaging techniques (*e.g.* electron microscopy) and DLS but is the forte of XPCS. In our current study, the spatial and temporal range probed by XPCS is 69.0 to 226.3 nm and 19 µs to 1.24 s due to limitation from beamline geometry and detector frame rate. In addition, each sample condition requires 12–24 h to accumulate sufficient statistics due to the very low scattering rate of CPMV, which rules out studies of non-equilibrium dynamics with the current coherent X-ray flux. However, we expect to break through these technical ceilings in the next few years with both next-generation X-ray sources that promise 100 times higher coherent X-ray flux (DLSRs and FELs) and the development of state-of-the-art, dedicated XPCS beamlines with high-speed, high-fidelity X-ray detectors. Combined with beamline automation, open-source software packages and rapid growth in artificial intelligence, our study will hopefully pave the road to guided self-assembly of emergent biomaterials, where the exact dynamic pathway of the biomacromolecules can be fine-tuned based on feedback from *in situ/operando* XPCS to produce bio- or bio-compatible materials with tailored properties.

## Supplementary Material

Supporting information detailing calibration of beam damage and calculation of virus radius. DOI: 10.1107/S1600577522008402/ay5603sup1.pdf
