# Supplementary material for: Direct measurement of Stokes–Einstein diffusion of Cowpea mosaic virus with 19 µs-resolved XPCS
Source: J Synchrotron Radiat. 2022 Oct 10;29(Pt 6):1429–35. doi: 10.1107/S1600577522008402 (PMC9641563; doi:10.1107/S1600577522008402)
Supplement: Supplementary file 1 [file s-29-01429-sup1.pdf]

JOURNAL OF SYNCHROTRON RADIATION

Volume 29 (2022)

Supporting information for article:

Direct Measurement of Stokes–Einstein Diffusion of Cowpea Mosaic Virus with 19 µs-resolved XPCS

Kacper Switalski, Jingyu Fan, Luxi Li, Miaoqi Chu, Erik Sarnello, Pete Jemian, Tao Li, Qian Wang and Qingteng Zhang

Supporting Information for "Direct Measurement of Stokes-Einstein Diffusion of Cowpea Mosaic Virus with 19 µs-resolved XPCS"

1. Determining CPMV Geometrical Radius R_0 from 1D SAXS

The calculation of 1D SAXS intensity was derived from T. Rieker *et al.*, Langmuir 1999, 15, 638-641. According to T. Rieker *et al.*, The scattering intensity for a colloidal suspension can be written as:

$$\mathrm{I(q)} \propto NS(q)P(q)$$

Where N is the number of colloidal particles, $P(q)$ is the form factor which describes scattering profile from individual particles, and $S(q)$ is the structure factor which describes the spatial distribution of the particles. For the CPMV suspension discussed in the main manuscript, at a weight fraction of 10 mg/mL and a dry density of 1.4 g/mL, the volume fraction of CPMV is 0.7%. With very dilute colloidal systems, S(q) approaches 1, leading to $\mathrm{I(q)} \propto P(q)$.

For dilute colloidal suspension with particle size distribution $D(R, R_0, \sigma)$, I(q) is expressed as:

$$\mathrm{I(q)} \propto \overline{P(q)} = \int_0^\infty P(q, a)\, D(a, a_0, \sigma)\, da$$

Here, a is the spatial scale of the colloidal particle, $D(a, a_0, \sigma)$ indicates the size distribution of the colloids. Specifically, for spherical particles with a Gaussian size distribution, we have:

$$P(q, R) = V^2 \left[\frac{\sin(qR) - \mathrm{qR}\cos(qR)}{(qR)^3}\right]^2$$

$$D(R, R_0, \sigma) = \frac{1}{\sigma\sqrt{2\pi}} \exp\left[-\frac{(R - R_0)^2}{2\sigma^2}\right]$$

Where R is the geometric radius of the particle, $V = \frac{4}{3}\pi R^3$ is the volume of the spherical particle, R_0 and σ are the mean and standard deviation of the Gaussian size distribution. Therefore:

$$\mathrm{I(q)} = I_0 \int_0^\infty R^6 \cdot \left[\frac{\sin(qR) - \mathrm{qR}\cos(qR)}{(qR)^3}\right]^2 \cdot \exp\left[-\frac{(R - R_0)^2}{2\sigma^2}\right] dR$$

Here I_0 is a scaling coefficient with the same unit as the absolute scattering cross-section I(q) determined from the experiment.

The integration was performed numerically using `scipy.integrate.quad` API from `scipy` library in Python. The lower and upper limit of the integration was set to 0 and 100 nm for realistic purposes. It was found that R_0 = 13.0 nm and σ = 1.2 nm provide the best fit to the experimental data as visually identified from Fig. 2 in the main manuscript.

2. Calibration of Radiation Damage

With the current coherent x-ray flux at station 8-ID-I of APS (1×10^{10} photons per second over a 10 μm × 10 μm spot), XPCS measurement on each CPMV sample condition (Fig. 3 and 4 in the main manuscript) requires ~10,000 repeats of 4 second measurements (2 second exposure and 2 second data transfer with x-ray shutter closed) that spans over 11 hours. However, the complete decorrelation of g_2 to baseline at longer delay times (Fig. 3) suggests that the collection of CPMV that are exposed to x-rays have completely moved out the beam by the end of each 2 second measurement the due to Brownian motion, so accumulative radiation damage is unlikely. As an additional precaution, each repeating measurement was taking at a spot separated by at least the size of the beam from the previous measurement.

For calibration of radiation damage within individual measurement, each 100,000-frame measurement is divided into sub-measurements containing a portion of the 100,000 frames, and the SAXS and XPCS analysis from the sub-measurements are checked for inconsistencies. Fig. S1 shows the 1D SAXS analyzed from every 10,000 frames over a single 100,000 frame measurement. Fig. S2 shows XPCS results from the first 50,000 frames (blue markers) and the second 50,000 frames (orange markers) and averaged over 14,328 repeats as in Fig. 3 of the main manuscript. No observable variation beyond the statistical fluctuation of the measurement has been observed in either SAXS and XPCS, indicating that radiation-induced effect does not contribute significantly to the conclusions derived from the measurement.

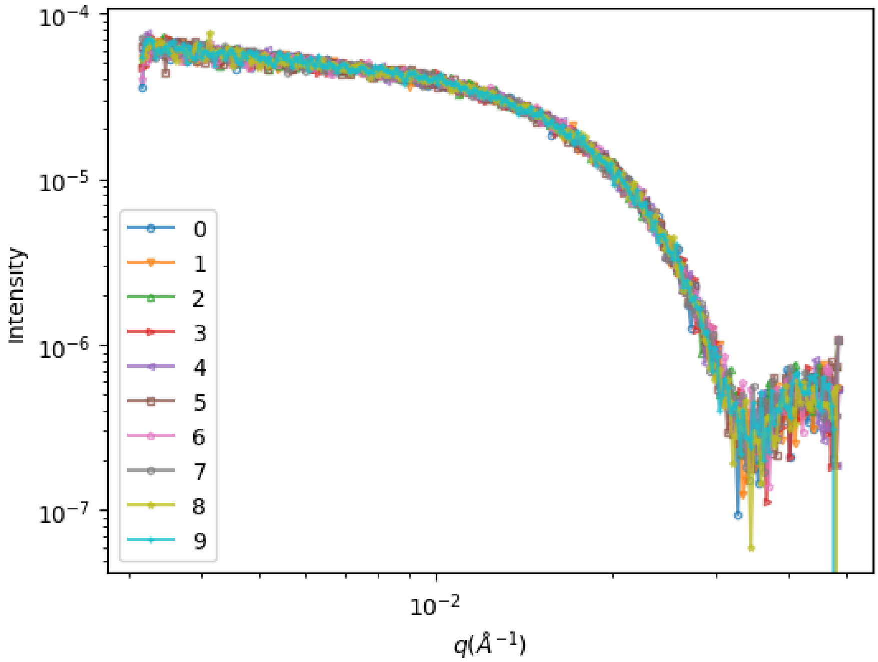

Fig. S1: SAXS from every 10,000 detector frames within a single 100,000-frame continuous acquisition at 52 kHz frame rate.

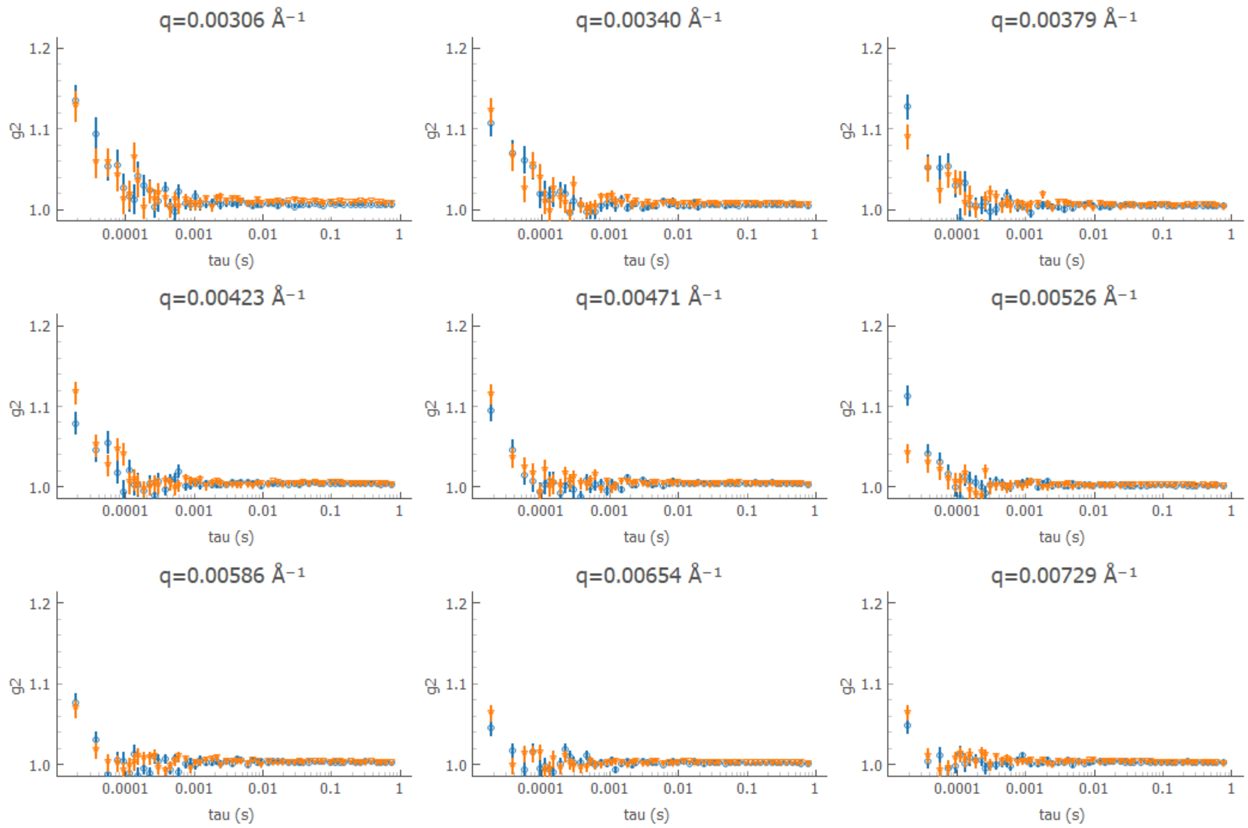

Fig. S2: Averaged XPCS results (14,328 repeats, Fig. 3) from the first 50,000 detector frames (blue) and the second 50,000 detector frames (orange) within 100,000-frame continuous acquisition.

3. *Multitau* Algorithm

Originally developed for DLS, *multitau* algorithm (Cipelletti and Weitz, Rev. Sci Instrum. **70** 3214 1999) is used in XPCS when the dynamics does not evolve significantly within the duration of the measurement, i.e., when the system is at equilibrium or when the system is evolving but the time scale of the evolution is much longer than the duration of the measurement. In these scenarios, the intensity autocorrelation function g_2 is assumed to be invariant of the measurement time t and only dependent upon the delay time τ between the frames. **Fig. S3** demonstrates this concept with a simplified data set containing 20 detector frames instead of 100,000 as in the manuscript. Here, $I_{i,j}(t)$ is the photon counts registered at pixel (i,j) at a given experimental time t. Time average of the multiplication of $I_{i,j}(t)$ with the translated version of itself, i.e., $I_{i,j}(t+\tau)$, yields $G2(\tau)$ (hence the term 'intensity autocorrelation'), while the time average of $I_{i,j}(t)$ and $I_{i,j}(t+\tau)$ yields $IF(\tau)$ and $IP(\tau)$. Binning $G2$, IF and IP over regions of interest (ROI) on the detector according to the ROIs used in the azimuthal average in Fig. 2, and then performing the division in Eq. 1 of the main manuscript, leads to $g_2(\tau, Q)$. This g_2 is further binned by approximately a factor of 10 in Q over larger ROIs to improve the statistics, as detailed in the main manuscript.

In *multitau*, assuming g_2 decays continuously over τ, the level of correlation at larger τ is insensitive to the fluctuations at smaller τ. As a result, for evaluation of g_2 at larger delay time τ, a recursive binning method is used, as demonstrated in **Fig. S4**. For an XPCS data set with N frames, with the recursive binning, the amount of computational resource required for both calculating and visualizing the dynamics scales with

$N \log_{10} N$ instead of N^2, which makes a tremendous difference if N is very large (e.g. 100,000). The recursive binning also significantly improves the statistics at larger τ.

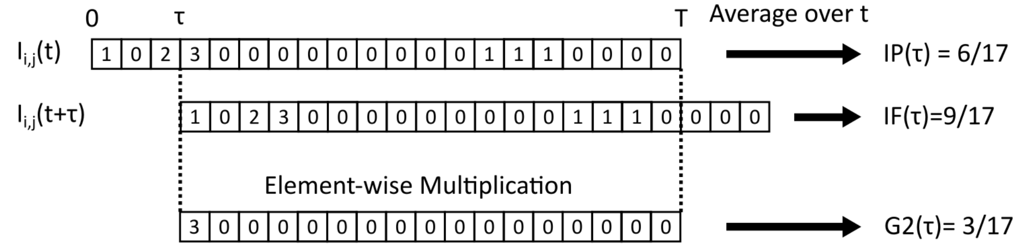

Fig. S3: Evaluation of IP, IF and $G2$ at pixel (i, j) from the time series of the intensity $I_{i,j}(t)$ based on Eq. 1 in the main manuscript. The dashed lines indicate the range of the time average. The maximum number of photon counts per frame at a pixel is 3 (bit depth of 2) for XSPA-500k at 52 kHz frame rate. In the event that a pixel registers 4 photons within a single frame, $I_{i,j}(t)$ rolls back to 0. However, since the probability of a correlation event involving an overflowed pixel is commensurate with $P1^5$ where $P1$ is the probably of a single photon event, for typical biological samples with a scattering intensity below 1×10^{-3} photon/pixel/frame at 52 kHz frame rate given the current level of coherent flux at 8-ID, the impact of count overflow on g_2 is negligible.

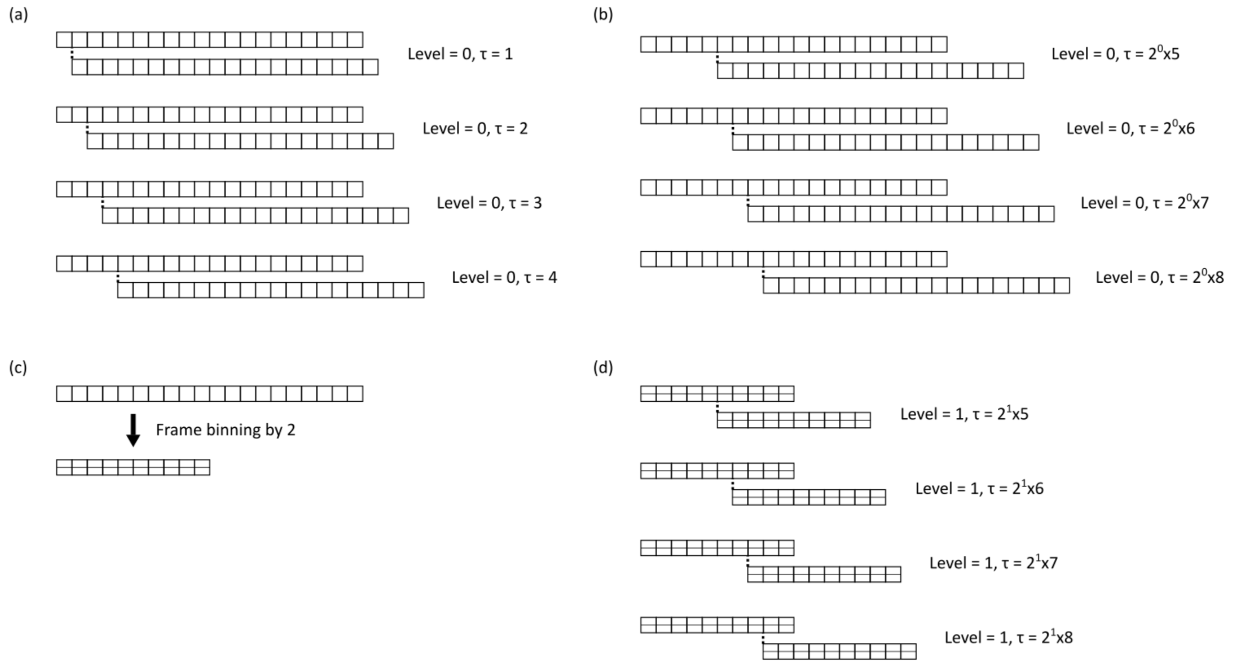

Fig. S4: Example of recursive binning method in *multitau* algorithm at $dpl = 4$ (delay per level). For $\tau \leq dpl$, no binning is required (**Fig. S4[a]**). For $\tau > dpl$, frame binning level starts from 0 and correlation is evaluated at $dpl + 1 \leq \tau \leq 2 \times dpl$ (**Fig. S4[b]**). After τ reaches $2 \times dpl$, the frames are binned by a factor of 2 (**Fig. S4[c]**) and the same process in **Fig. S4[b]** is repeated with binned frames (**Fig. S4[d]**). This iterates till τ becomes larger than the total length of the binned frame sequence.
